# Q&A: Array tomography

**DOI:** 10.1186/s12915-018-0560-1

**Published:** 2018-09-06

**Authors:** Stephen J Smith

**Affiliations:** Allen Institute for Brain Science, Seattle, WA USA

## Abstract

Array tomography encompasses light and electron microscopy modalities that offer unparalleled opportunities to explore three-dimensional cellular architectures in extremely fine structural and molecular detail. Fluorescence array tomography achieves much higher resolution and molecular multiplexing than most other fluorescence microscopy methods, while electron array tomography can capture three-dimensional ultrastructure much more easily and rapidly than traditional serial-section electron microscopy methods. A correlative fluorescence/electron microscopy mode of array tomography furthermore offers a unique capacity to merge the molecular discrimination strengths of multichannel fluorescence microscopy with the ultrastructural imaging strengths of electron microscopy. This essay samples the first decade of array tomography, highlighting applications in neuroscience.

**Electronic supplementary material:**

The online version of this article (10.1186/s12915-018-0560-1) contains supplementary material, which is available to authorized users.

## What is array tomography?

Array tomography (AT) is a versatile microscopy method that offers superlative opportunities to explore cell and tissue architectures in three dimensions. It is well suited to seamless imaging of large tissue volumes in extremely fine structural and high molecular detail, positioning the method nicely for emerging post-transcriptomic tissue biology applications. A fluorescence microscopy AT mode (FM-AT) delivers volumetric resolution and molecular marker multiplexing highly superior to traditional fluorescence microscopies, while an electron microscopy AT mode (EM-AT) readily captures three-dimensional ultrastructure at size scales that would require prohibitive effort using traditional serial-section EM methods. Where AT is entirely unique, however, is in supporting a “voxel-conjugate” combination of the fluorescence and electron microscopy modes (FM/EM-AT), where three-dimensional light and electron images are acquired in essentially perfect volumetric register. These attributes establish AT as an ideal choice for the most demanding analyses of diverse cellular architectures within mature and developing tissues, including brain. This essay will draw examples mainly from neuroscience, but AT methods are also finding many cell and tissue biology applications outside of neuroscience [1–13].

**Fig. 1:**
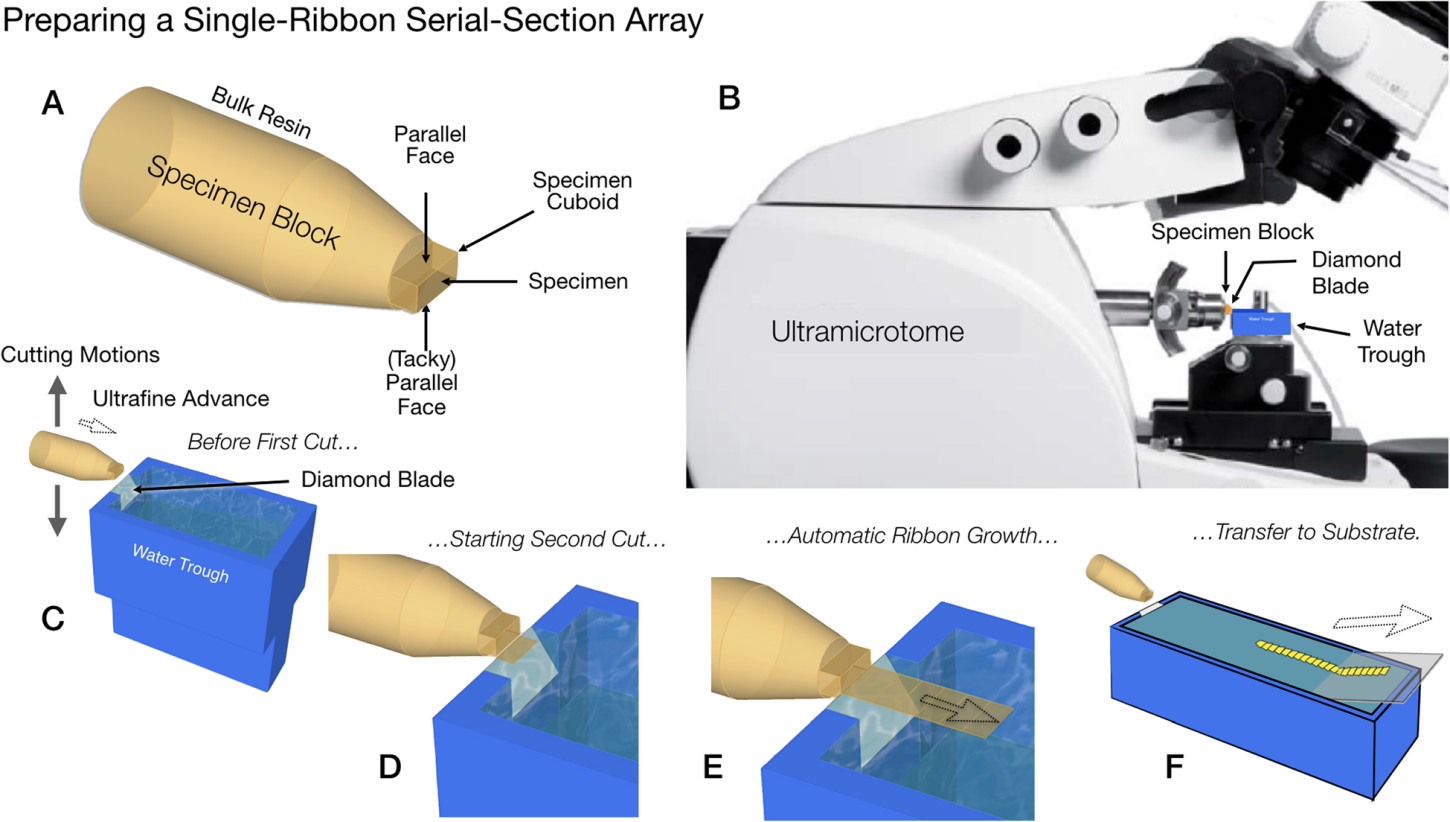
One simple form of tomography array production. The specimen is fixed and embedded in an acrylic resin. The resulting resin block (**a**) is then trimmed to orient the embedded specimen for sectioning on an ultramicrotome (**b**). The standard ultramicrotome action automatically produces a ribbon of serial ultrathin sections on a water surface (**c**–**e**). The ribbon is then transferred to the surface of a microscope coverslip (**f**) or other solid material. Different array production methods place multiple ribbons or multiple individual sections onto varied solid substrates

**Fig. 2:**
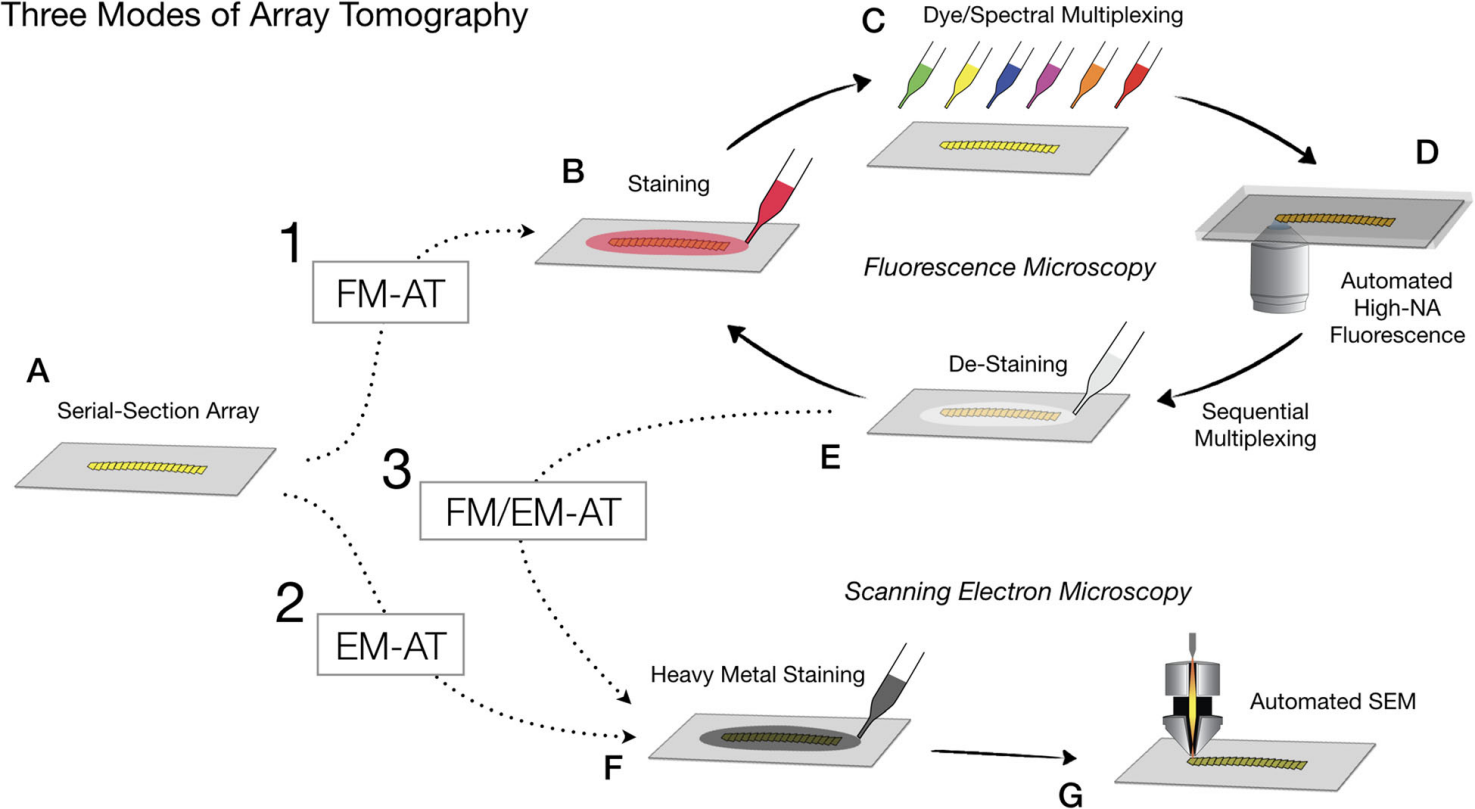
Alternative fluorescence (FM-AT), electron (EM-AT) and combined (FM/EM-AT) modes of array tomography. An array of serial ultrathin sections (**a**) (e.g., a single-ribbon coverslip produced as in Fig. 1) may be stained and imaged for multiplex fluorescence microscopy (*dotted arrow 1*), scanning EM (*dotted arrow 2*), or both (*dotted arrows 1 and 3*). (**b**–**e**) Schematizes possible combinations of spectral and sequential fluorescence multiplexing modes. (**f**, **g**) Schematizes (optional) array staining and image acquisition for electron microscopy

**Fig. 3:**
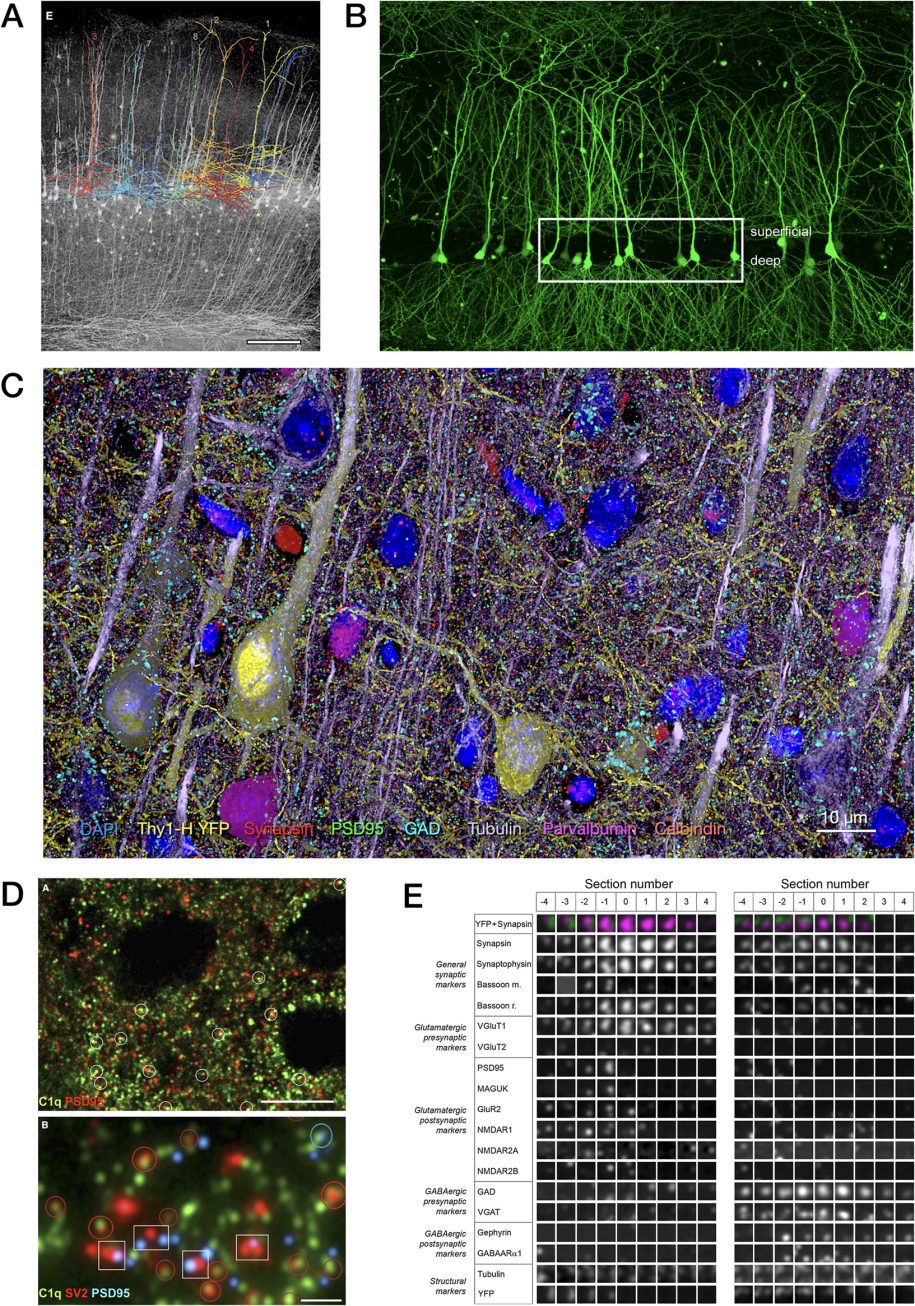
Fluorescence array tomography (FM-AT) images of mouse cortex representing the superior volume field size, resolution, and multiplex capabilities characteristic of this modality. **a** Thy1-YFP line H barrel cortex pyramidal cells, with eight superimposed dendrite tracings (from Fig. 2 in [28]). **b** CA1 hippocampal cortex pyramidal cells from Thy1-EGFP line M mouse (from Fig. S2 in [27], Copyright (2016), with permission from Elsevier). **c** Layer 5 barrel cortex in Thy1-YFP line H mouse illustrating results of sequential+spectral multiplexing of the eight molecular markers indicated in barrel cortex (unpublished data courtesy of KD Micheva). **d** Synaptic localization of C1q in developing mouse LGN thalamus (from Fig. 4 in [101], Copyright (2007), with permission from Elsevier). **e** Synaptograms of excitatory (left) and inhibitory (right) synapses illustrating sequential+spectral multiplexing of 18 molecular markers (from Fig. 6 in [37], Copyright (2010), with permission from Elsevier)

**Fig. 4:**
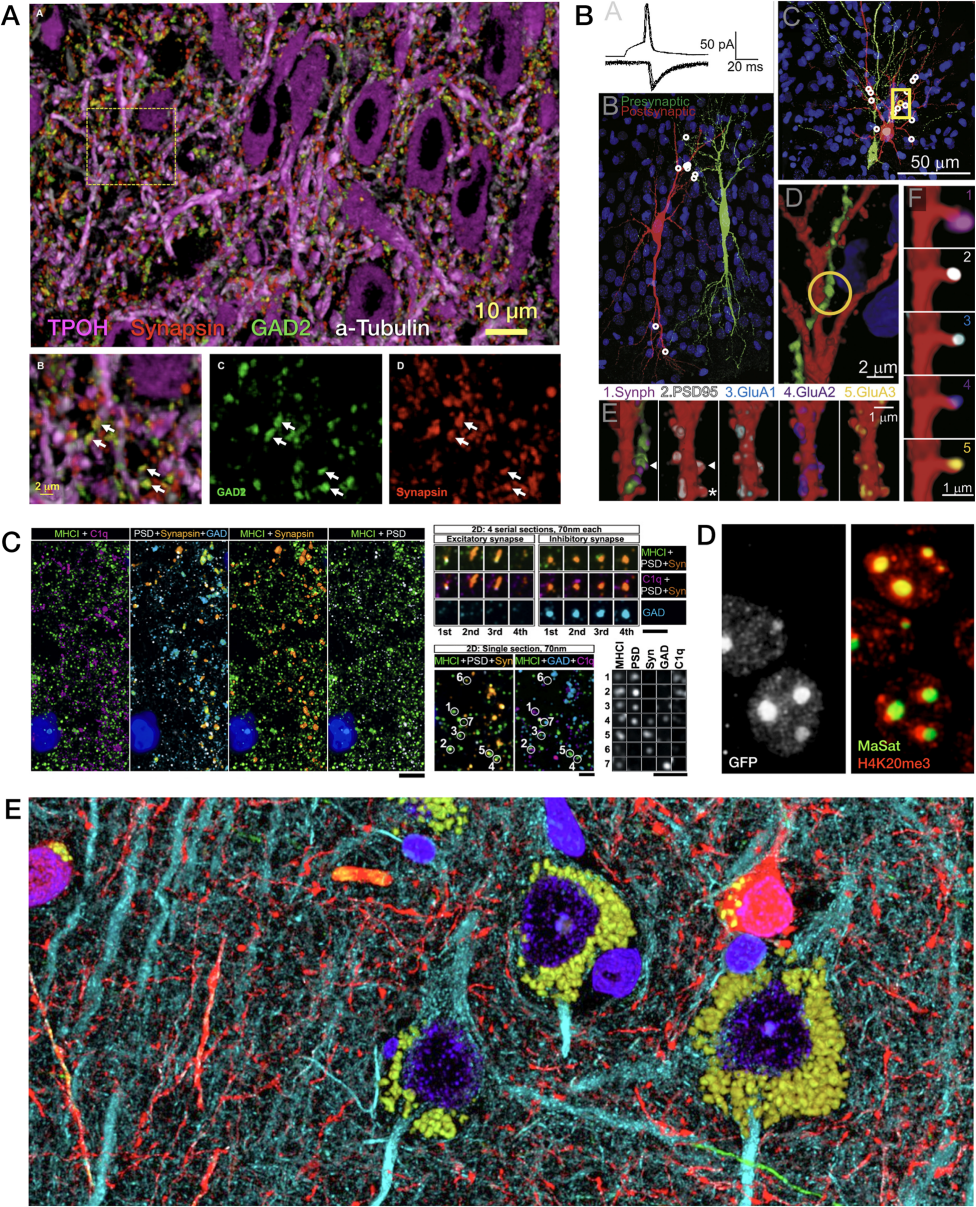
FM-AT images from varied regions of mammalian brain sampling the wide range of neuroscience FM-AT applications to date. **a** Synaptic anatomy of the dorsal raphe nucleus (from Fig. 2 in [102]). **b** Mechanistic analysis of excitatory synaptic transmission and synaptic plasticity in CA3 hippocampus (from Fig. 3 in [97], Copyright (2016), with permission from Elsevier). **c** Synaptic localization of MHCI proteins in mouse lateral geniculate nucleus at P7, during retinogeniculate critical period (from Fig. 4 in [98], Copyright (2009), with permission from Elsevier). **d** Correlated immunofluorescence/DNA-FISH images showing expansion of H4K20me3 histone modification into pericentromeric heterochromatin in Mecp2-null (*GFP-*) but not control (*GFP+*) nuclei in a mosaic Rett syndrome model mouse (from Fig. 4 in [56], Copyright (2015), with permission from Elsevier). **e** Human neocortex illustrating clear imaging of brightly autofluorescent lipofuscin granules (*yellow*) without out-of-focus flare obscuration of nearby cellular features (*blue*, DAPI; *red*, GABA; *cyan*, tubulin; *green*, neurofilament heavy chain). (Unpublished data courtesy Kristina Micheva)

Various implementations and applications of AT are described in detail by excellent recent reviews [1–3, 6, 14–16]. Features common to all AT implementations include: (A) physical ultrathin serial sectioning of a fixed, resin-embedded specimen, (B) collection of the resulting serial sections to form an array on a solid substrate, (C) staining and digital imaging of the resulting serial section array by fluorescence microscopy (FM-AT) and/or electron microscopy (EM-AT), and (D) computational stitching of the resulting two-dimensional image tiles into coherent volumetric images. Figure 1 illustrates one simple approach to tomography array fabrication. Careful trimming and preparation of the specimen block [17] and the repetitive cutting action of a standard diamond-knife ultramicrotome results in the automatic production of a continuous “ribbon” of serial sections on a water surface. The serial-section ribbon is then readily transferred to a solid substrate such as an optical coverslip. Figure 2 schematizes the three major modes of AT, using a single-ribbon section array as an example. It also schematizes FM-AT support for both spectral and sequential modes whenever it is desired to multiplex large numbers of fluorescence markers. Imaging results exemplifying key strengths of each AT mode are compiled in Figs. 3 and 4 (FM-AT), 5 (EM-AT), and 6 and 7 (FM/EM-AT) and in supplemental video materials in Additional files 1, 2, 3.

**Fig. 5:**
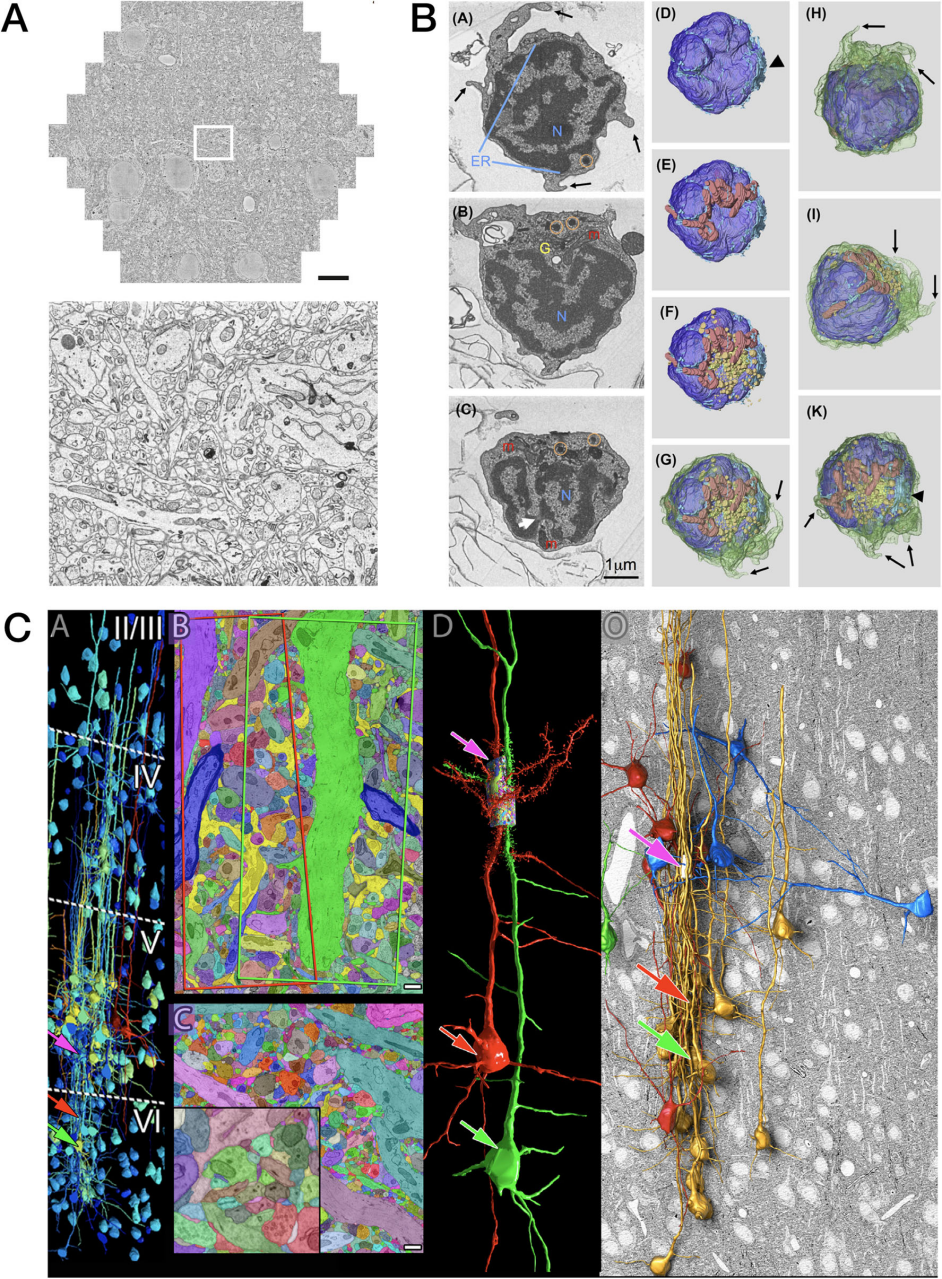
Electron array tomography (EM-AT) images representing the excellent EM image quality, large field sizes, and amenability to volume object segmentation characteristic of this modality. **a** Demonstration of excellent results from multibeam SEM imaging of a single array section on carbon nanotube tape substrate, where lower panel magnifies one region from the very large single multibeam field (scale bar 10 μm) in *upper panel* (from Fig. 7 in [29]). **b** Reconstruction of a zebrafish immune cell to create an inventory of organelles (from Fig. 2 in, *ref* [7]). **c** Selected results from multiscale reconstruction of a small volume of mouse cortex (from Fig. 3 in [55], Copyright (2015), with permission from Elsevier)

**Fig. 6:**
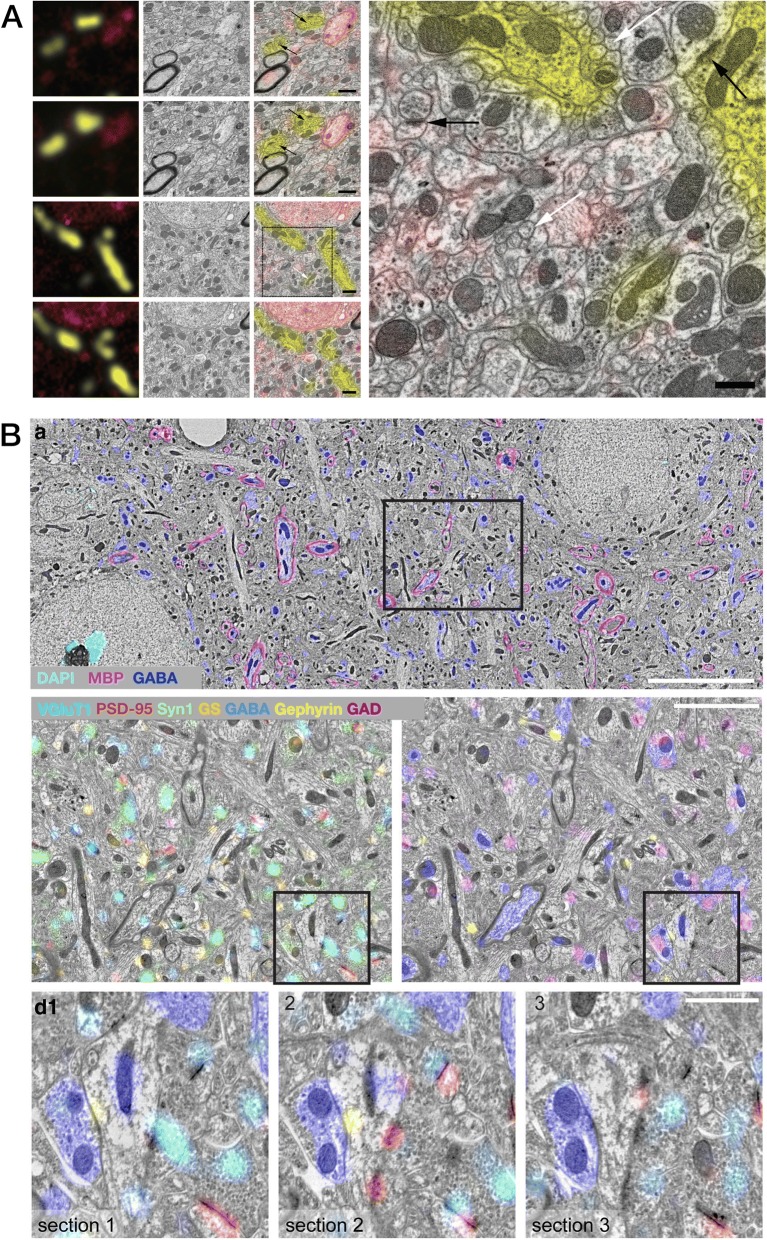
Fluorescence / Electron Array Tomography (FM/EM-AT) images representing the unique capacity of this modality to combine fluorescence and electron imaging in volumetric register (part 1/2). **a** Co-registration of FM-AT and EM-AT images for songbird brain projectomics (from Figs. 3 and 4 in [30]). **b** Molecular multiplexing via voxel-conjugate FM/EM-AT for synaptomic analysis of mouse somatosensory cortex (from Fig. 5 in [31])

**Fig. 7:**
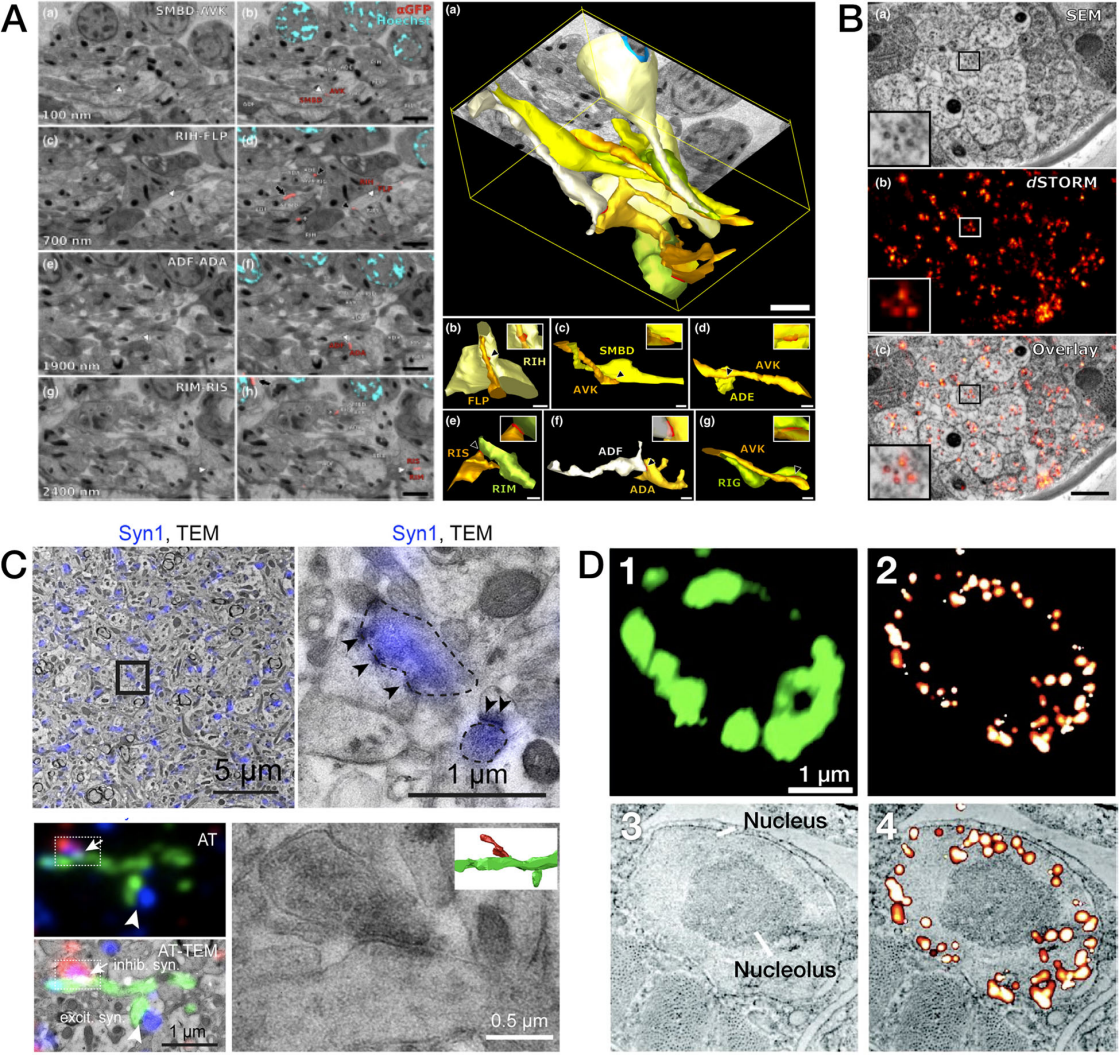
Fluorescence/electron array tomography (FM/EM-AT) images (part 2/2). **a** FM/EM-AT-derived three-dimensional models of neuronal gap-junctional connectivity in *C. elegans* (from Fig. 6 in [32]). **b** Microtubules imaged by EM-AT correlated with STORM FM-AT in a single array section of *C. elegans* ventral nerve cord (from Fig. 8 in [32]). **c** Rigorous identification of synaptic connection in mouse hippocampus by correlative AT-TEM (from Fig. 1 in [27], Copyright (2016), with permission from Elsevier). **d** Localization of a histone H2B fusion protein in a single section of a *C. elegans* muscle cell nucleus. *D1* Summed TIRF fluorescence image; *D2* PALM fluorescence image; *D3* Backscattered-electron SEM image; *D4* Overlay of PALM and SEM images (from Fig. 1 in [44], reprinted with permission from Springer Nature, Copyright (2010))


**Additional file 1:** Movie S1. Fluorescence (FM-AT) and electron (EM-AT) images of a single mouse cortex array section, overlaid in pixel-precise register illustrating combination of multichannel FM of a large area (approximately 0.4 × 1.0 mm, spanning all six cortical layers) with EM sampling of a smaller subfield (unpublished Allen Institute data). (Procedures as schematized in arrow track 3, Fig. 2. The rationale for registering a large FM-AT field with a much smaller EM-AT field is discussed in the text section “What limits AT specimen size”). In this video rendering, the field of view gradually zooms 200-fold into a very small subfield in layer 5. At the higher zooms, it is evident that synaptic protein (PSD95, GluN1, VGluT1, Synapsin, GAD2 and Gephyrin), nuclear DNA (DAPI), myelin (MBP), and GABA markers align with EM images as expected from current biological models of mammalian cortex and synapses. Colors representing ten channels of molecular fluorescence are modulated periodically in this video to better accommodate the limitation of human color vision to (at most) three discrete color channels. The specimen samples VISp cortex of a transgenic mouse in which expression of a fluorescent protein (TdTomato) was driven mainly in layer 4 pyramidal cells [103] (MOV 117816 kb)



**Additional file 2:** Movie S2. Visualization of an eight-channel FM-AT volume of mouse somatosensory cortex illustrating: **a** results of high-order sequential marker multiplexing (as schematized in Fig. 2 and discussed text sections “What makes AT especially suitable for sequential multiplexing”); and **b** the high volumetric resolution of FM-AT (as discussed in “What limits FM-AT resolution” section). This volume (h, w, d = 130 × 90 × 3.5 um) was sampled from a Thy1-H-YFP transgenic mouse (unpublished data courtesy of Kristina Micheva). Specimen and all methods are as previously described [37]. Eight colors representing synaptic (Synapsin, PSD95, GAD), neuron type (Thy1-H, parvalbumin, calbindin), and organelle (DAPI, tubulin) markers are modulated in separate groups to accommodate limitation of human color vision to three discrete channels (MOV 104130 kb)



**Additional file 3:** Movie S3. “Machinery of Mind” renders an FM-AT volume image of mouse somatosensory cortex to evoke the beautiful intricacies typical of the synaptic networks that endow all animal nervous systems with their astonishing functional capabilities. This video is best appreciated when played with sound (original musical score and performance by Catherine Rose Smith) over a full-range audio system or good headphones, turned up loud. This is a revised version of supplemental video #1 from Micheva, et al. [37] (Copyright (2010), with permission from Elsevier), enhanced by the addition of a new prolog sequence, based on an MRI atlas dataset [104] to indicate the relationship of the FM-AT volume sampled (approximately 1.5 mm by 0.5 mm wide by 12 μm deep) to the whole mouse brain. A subset of glutamatergic neurons (mostly layer 5 pyramidal cells) are rendered in *green*, reflecting expression of YFP in the Thy-1H-YFP mouse from which the specimen was obtained. The *red* puncta evident in this FM-AT visualization correspond to individual synapses: approximately eight million can be resolved within the rendered volume. The *blue filaments* represent microtubule bundles and are visible mainly in the dendrites of non-YFP expressing neurons. Details of the FM-AT volume capture are as described in [37]. Briefly, the FM-AT volume data were captured by imaging a single ribbon of 60 serial sections, each cut 200 nm thick from a block of LR-White embedded tissue and labeled by post-embedding immunostaining. The FM-AT volume extends from the pial surface through all six layers of cortex and subcortical white matter into the striatum. This dataset comprises 3.7 billion voxels and three color planes (each one byte wide: YFP, *green*; anti-Synapsin I, *red*; anti-alpha-tubulin, *blue*). (MOV 579726 kb)


## Why is it called “array tomography”?

The term “array tomography” was introduced by a 2007 neuroscience publication [17], but earlier and contemporaneous writings presage individual fluorescence and electron microscopy elements of AT [18–22]. The terminology is straightforward: “array” refers to arrangement of serial sections in spatial array on a planar solid surface, while “tomography” alludes to the capture of three-dimensional structure from two-dimensional image “slices” (fr. Greek “*tomos*”, slice). Confusion may result, however, from the widespread use of “computed axial tomography” (CAT) in reference to a form of three-dimensional X-ray imaging widely used in clinical radiology, and of “electron tomography” (ET) in reference to an ultra-high-resolution form of three-dimensional electron microscopy. For both CAT and ET, volumetric image “slices” are computed from projection images acquired from multiple angles and no physical slicing is usually involved [23, 24]. Unlike CAT and ET, AT does not generally involve transforming rotational projections.

As defined above, AT might conceivably apply to all forms of serial-section microscopy, including serial-section transmission electron microscopy (ssTEM) [25, 26]. The AT terminology is restricted here, however, to arrays placed on stable solid substrates such as glass coverslips, flexible tape, or silicon wafers, as opposed to the open grid slots or delicate, ultrathin electron-transparent support films required for ssTEM imaging. Physical stability of the array substrate is essential to several distinctive AT benefits, such as specimen stability during the repeated solution changes necessary for sequential multiplexing. As electron absorption by a substantial solid substrate precludes easy use of transmission electron microscopy (TEM), scanning electron microscopy (SEM) is most often used for EM-AT because backscattered or secondary electron images can be acquired in a reflection mode. Though TEM offers the ultimate in electron microscopic resolution, SEM resolution is more than adequate for a wide range of cell and tissue science applications. That said, one recently introduced hybrid variant of AT, called AT-TEM, uses a film transfer method to conjoin the stability advantages of fluorescence AT imaging on a solid substrate with the resolution and speed advantages of subsequent TEM imaging [27, 28].

## When should one consider using AT?

The use of FM-AT (Figs. 2, 3 and 4) should be considered for volumetric fluorescence imaging of fixed tissue specimens whenever there is need for very high resolution, high-order molecular multiplexing and/or rigorously depth-independent quantification of fluorescence signal intensities. Use of EM-AT (Figs. 2 and 5) offers perhaps the most convenient approach to volumetric electron microscopy available. Use of FM/EM-AT (Figs. 2, 6 and 7) offers unique opportunities to combine the molecular discrimination strengths of fluorescence microscopy with the unrivaled structural resolution of electron microscopy [1, 2, 6, 11, 14, 15, 17, 27–37]. While other approaches to correlative light and electron microscopy of individual specimens have proven extremely useful [38], AT alone offers a path to perfect registration of fluorescence and SEM voxels over extended volumes. The unique ability of conjugate FM/EM-AT to unify molecular and ultrastructural views of neural network architectures sets a standard for the emerging fields of synaptomics and connectomics. The analysis of diverse cortical synapse populations (as illustrated in Figs. 3, 4, 5, 6 and 7) provides examples of applications benefitting from each of the major AT strengths.

AT confers an additional special advantage for quantitative fluorescence imaging of brain specimens from older animals (including all adult humans), which are often suffused with brightly autofluorescent lipofuscin deposits. With standard fluorescence microscopy methods, out-of-focus flare from lipofuscin usually obscures nearby tissue features and confounds fluorescence interpretation and quantification [39]. The extremely high Z-axial resolution resulting from AT ultrathin physical sectioning eliminates such lipofuscin interference decisively (Fig. 4e) and may enable improved fluorescence analysis of the intimate cellular milieu in which lipofuscin forms in aging brains.

## How are specimens prepared?

By definition, all forms of AT require serial sections to be cut and transferred to a solid substrate for imaging. Cutting the very thin sections necessary for high-resolution AT requires in turn that tissue first be fixed, dehydrated, and embedded in a hard, cross-linked polymer resin matrix. Years of transmission electron microscopy (TEM) and immuno-TEM practice have resulted in the development and optimization of a variety of alternative fixation, dehydration, and embedding materials and methods [40], but it is necessary to consider various tradeoffs in choosing amongst these alternatives.

Mild chemical fixation, e.g., by formaldehyde alone, is generally preferable for preservation of immunoreactivity but compromises preservation of fine ultrastructural details. More stringent chemical fixatives, e.g., glutaraldehyde and/or osmium, better preserve ultrastructure but compromise immunoreactivity. Dehydration methods also strongly influence tradeoffs between preservation and immunoreactivity, with freeze substitution methods (where the specimen is frozen and water is replaced by organic solvent at very low temperatures) generally yielding superior results but requiring more complex procedures and equipment in comparison with room-temperature solvent replacement methods. The choice of embedding resin chemistry also entails a tradeoff, with acrylic embedding resins (such as LR White and Lowicryls) offering much better antibody access for immunofluorescence while epoxy resins generally yield superior EM image quality. While ultrathin sections can be cut by resin-free cryosectioning methods, which might avoid structure–immunoreactivity tradeoffs, no applications to AT have yet appeared, reflecting the imposing difficulty of serial cryosection production and collection. Each of the other fixation, dehydration, and resin-embedding methods mentioned here has been employed for AT, with choices amongst these diverse methods being driven primarily by the project goals and tradeoffs outlined above [5, 17, 31, 41–43]. In choosing a tissue preparation method for any specific AT application, the established literatures from electron and correlative light/electron microscopy practice will provide much additional useful guidance [38, 40, 44–48].

## How are arrays constructed?

The diamond-knife ultramicrotome, refined over many decades of use for transmission electron microscopy (TEM; Fig. 1b), accomplishes the basic, automated cutting of ultrathin serial sections easily and reliably. All forms of AT described so far employ this standard instrument, which automatically cuts either individual, free-floating sections or continuous “ribbons” of serial sections (Fig. 1c–e) onto the surface of water held in a small pool just behind the diamond knife edge. These very thin and delicate sections or ribbons must then be transferred from the water surface to a solid substrate. Simple manual means, usually involving an “eyelash” tool to move section ribbons on the water surface, have sufficed for such transfer in small-scale projects, but applications requiring the imaging of larger tissue volumes, such as those required for the analysis of extended synaptic networks, have motivated development of higher-throughput, automated means of water-to-solid section transfer as necessary to build large-scale arrays.

**Fig. 8:**
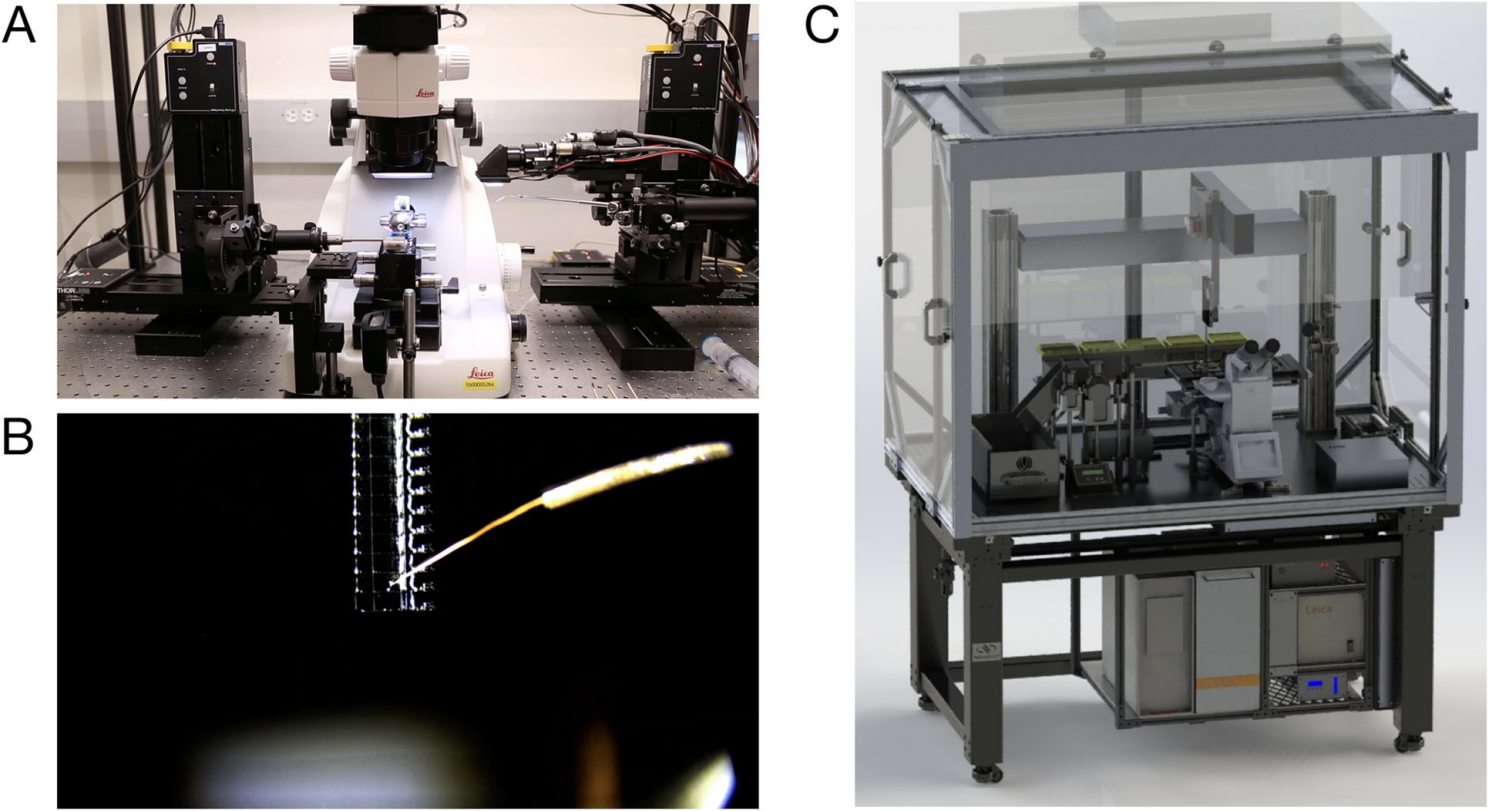
Automated array construction and high-throughput fluorescence imaging. **a** “ArrayBot”, based on a standard ultramicrotome, combines ten computer-controlled motion axes with machine vision camera to automate the most critical steps in serial array construction and facilitate construction of large sets of contiguous single-ribbon arrays. **b** Robotically manipulated deer hair positions serial-section ribbon on water surface in ArrayBot trough. **c** “Robofluidic” AT fluorescence microscope automates staining and image acquisition across multiple single-ribbon serial-section arrays. This microscope is optimized for image acquisition speed via intense laser illumination, fast mechanics, and tight control timing. Both were developed at the Allen Institute to facilitate high-throughput, large-scale array tomography

Varying degrees of section collecting automation have been introduced to accommodate volumes requiring more than a few dozen serial sections—up to many thousands of sections. Hayworth and colleagues [41, 49] introduced a robust tool that automates the collection of individual sections for EM-AT. This “automated tape-collecting ultramicrotome” (ATUM) is now commercially available [50] and a novel fluorescence-compatible tape material now also permits the use of the ATUM for FM-AT and FM/EM-AT [29]. Specialized devices to ease collection of section ribbons onto rigid substrates are described in publications [51, 52] or commercially available [53] and https://www.leica-microsystems.com/array-tomography/. The Allen Institute has developed “Arraybot” collectors that use multiple, computer-controlled motion axes to automate handling of glass coverslips and placement of serial-section ribbons (Fig. 8a). Another potentially revolutionary new array production process, based on magnetic guidance of serial sections onto the array substrate, has also been reported [54]. The tape collecting method is presently the most mature and is highly amenable to pure EM-AT applications. The Arraybot collectors method may be preferred when the superior optical qualities of the optical coverslip substrate are desired to support the highest resolution fluorescence imaging in the FM-AT or FM/EM-AT modes.

A variety of materials have been used as AT array substrates. Early AT substrates were traditional “subbed” histology slides—standard glass microscope slides coated with a layer of hardened gelatin to promote section adhesion [17]. While serviceable for less demanding AT applications, this substrate is limited by less-than-ideal optical properties, mediocre section adhesion, and instability under the electron beam. Improved optics can be obtained by adhering the sections or ribbons directly to a precision coverslip, rather than onto a slide, but the gelatin layer remains problematic. Much improved FM-AT and EM-AT image quality and array stability are now obtained by replacing the gelatin with a thin, transparent layer of evaporated carbon laid down upon a silanized coverslip surface [31]. Flexible polymer substrates, such as carbon-coated Kapton tape, have enabled the use of a simple tape-transport method for automated, high-throughput array section collection [2, 10, 29, 49, 55]. Continuous tapes of array sections may be readily cut into shorter segments and glued to small silicon wafers in multi-row arrays for SEM imaging. Other electron and fluorescence AT methods involve direct adhesion of array sections to silicon wafer surfaces [6, 30].

## How are arrays stained?

FM-AT signals may result from immunofluorescence labeling, dye injection, or transgenically expressed fluorescent proteins [6, 17]. Signals from DNA FISH have also been demonstrated [56]. Immunolabeling may be accomplished either prior to resin embedment (pre-embedding) or after embedding and sectioning (post-embedding). Pre-embedding immunolabeling generally offers higher immunolabeling efficiency near the specimen surface, but at the cost of strongly depth-dependent labeling efficiency. Post-embedding labeling (performed after ultrathin sectioning) offers depth-insensitive labeling, while sacrificing molar labeling efficiency and thereby signal-to-noise ratio. Post-embedding immunostaining may also be preferred because of superior preservation of ultrastructure [57] and superior sequential multiplexing possibilities. The minimal thickness of AT sections greatly facilitates quick and reliable specimen staining by eliminating the need for staining molecules to percolate via binding-restricted diffusion into a thick tissue section. It should be noted, however, that some antibodies that work well for pre-embedding staining do not work as well in post-embedding applications (including AT), presumably because specimen dehydration and resin embedding alter or hide target protein epitopes [40, 58]. It is, therefore, generally advisable to search any available resources [17, 31, 37, 59–61] regarding antibodies that have established efficacy in post-embedding or AT applications, or otherwise be prepared to test multiple antibodies for suitability to such specimens [62].

For EM-AT, contrast is usually generated by staining cellular membranes and proteins non-specifically with heavy metals such as osmium, lead, or uranium. Again, staining may be accomplished either pre-embedding or post-embedding, but pre-embedding metal staining can be considered only when no subsequent immunolabeling is intended, as the methods are incompatible. Pre-embedding metal staining can provide excellent results with small tissue samples, but results are often inconsistent with larger specimens. Post-embedding staining for EM-AT avoids any depth-dependent stain variations and can be carried out following the conclusion of FM-AT imaging, providing the essential basis for conjugate FM/EM-AT image acquisition.

## How are arrays imaged?

FM-AT images may be acquired by standard widefield fluorescence microscopy, by confocal fluorescence microscopy, or by lateral super-resolution modes such as PALM, STORM, STED, or structured illumination. The choice of fluorescence microscopy mode is influenced by tradeoffs between achievable lateral resolution, imaging speed, and the number of fluorescence “color” channels accessible in a single stain-image-wash round. Straight widefield fluorescence offers a very attractive combination of low instrument cost, very high acquisition speed, high channel capacity, and high resolution, truly diffraction-limited even when using aberration-prone high NA objectives (as explained in the following section). When sufficiently high fluorescent label density is achievable (e.g., [3, 22, 44, 63]), the lateral super-resolution AT modes offer still higher lateral resolution, though this advantage comes at present with substantial costs in process complexity and speed.

Scanning electron microscopy (SEM) captures EM images for EM-AT and FM/EM-AT. While simpler tungsten-filament SEMs might be used for this purpose, the improvements in acquisition speed and effective resolution with the more complex (and, unfortunately, expensive) field-emission SEM (FE-SEM) are dramatic. Though FE-SEM image acquisition is fast in comparison to tungsten SEMs, it is still very slow, however, in comparison to fluorescence image acquisition on a volume basis. A recently introduced multi-beam SEM (mSEM) promises enormous increases in SEM acquisition speed, though this advantage entails further very large increases in instrument complexity and cost.

For both FM-AT and EM-AT modes, computational automation of microscope mechanical axes and image acquisition is extremely helpful and becomes a virtual necessity when it is desired to image large specimen volumes, where many thousands of multispectral image tiles must be acquired. Focus (and in the case of SEM, stigmation of the electron beam) must be automated and XY stage motors must advance the field of view automatically to image many sections in sequence. When imaging larger specimen volumes, it is generally necessary to extend the microscope’s limited two-dimensional field of view by lateral mosaic stitching of multiple image fields. The automation of multichannel fluorescence acquisition is often accomplished by motorizing filter changers, although some newer solid-state light sources and multi-band filter sets allow faster channel selection by switching excitation bands without moving parts. A fluorescence microscope optimized at the Allen Institute for high-throughput, highly automated staining and imaging of AT arrays is depicted in Fig. 8c. Commercial availability of hardware and software specialized for all AT image acquisition modes now promises to open these powerful but complex AT methodologies to much wider adoption [50, 53, 64, 65].

## What limits FM-AT resolution?

Fluorescence AT readily yields volumetric resolution much higher than whole-mount, diffraction-limited fluorescence methods, such as wide-field or confocal microscopy. The AT improvement in resolution begins with the fact that AT physical sections are usually much thinner (40–100 nm) than the diffraction limit along the focal (Z) axis (theoretically > 500 nm, even at the highest NAs, but usually further worsened in tissue whole-mounts by optical aberrations). The volumetric resolution of FM-AT thus immediately improves by a factor of 5–10 over the Z-axis diffraction limit. This is significant since Z-axis resolution is always much worse than lateral resolution, and is therefore the Achilles’ heel of normal fluorescence resolution.

Lateral (X-Y) FM-AT resolution is also improved substantially in comparison to whole-mount fluorescence, due to minimization of optical aberrations that otherwise compromise lateral resolution in thick specimens. Since AT sections are very thin and placed exactly at the surface of a precision optical coverslip, the stringent conditions required for truly diffraction-limited resolution by a high-NA oil objective are met exactly [66]. With thicker specimens, resolution-robbing aberrations due to optical inhomogeneities in specimen or mounting medium are very difficult to avoid. Moreover, since array sections are much thinner than the optical depth of focus, noise contributions from out-of-focus specimen elements are completely eliminated. These factors dramatically improve image quality and permit application of the most precise two-dimensional deconvolution methods for optimal two-dimensional image restoration [67]. These reductions in optical aberrations and image noise may boost effective lateral resolution for AT by at least a factor of two in comparison to typical whole-mount fluorescence imaging. These same improvements in basic imaging optics confer substantial benefits even when using super-resolution array imaging methods such as PALM, STORM, or STED, because these methods still benefit in speed and limiting resolution from truly diffraction-limited optics [68].

**Fig. 9:**
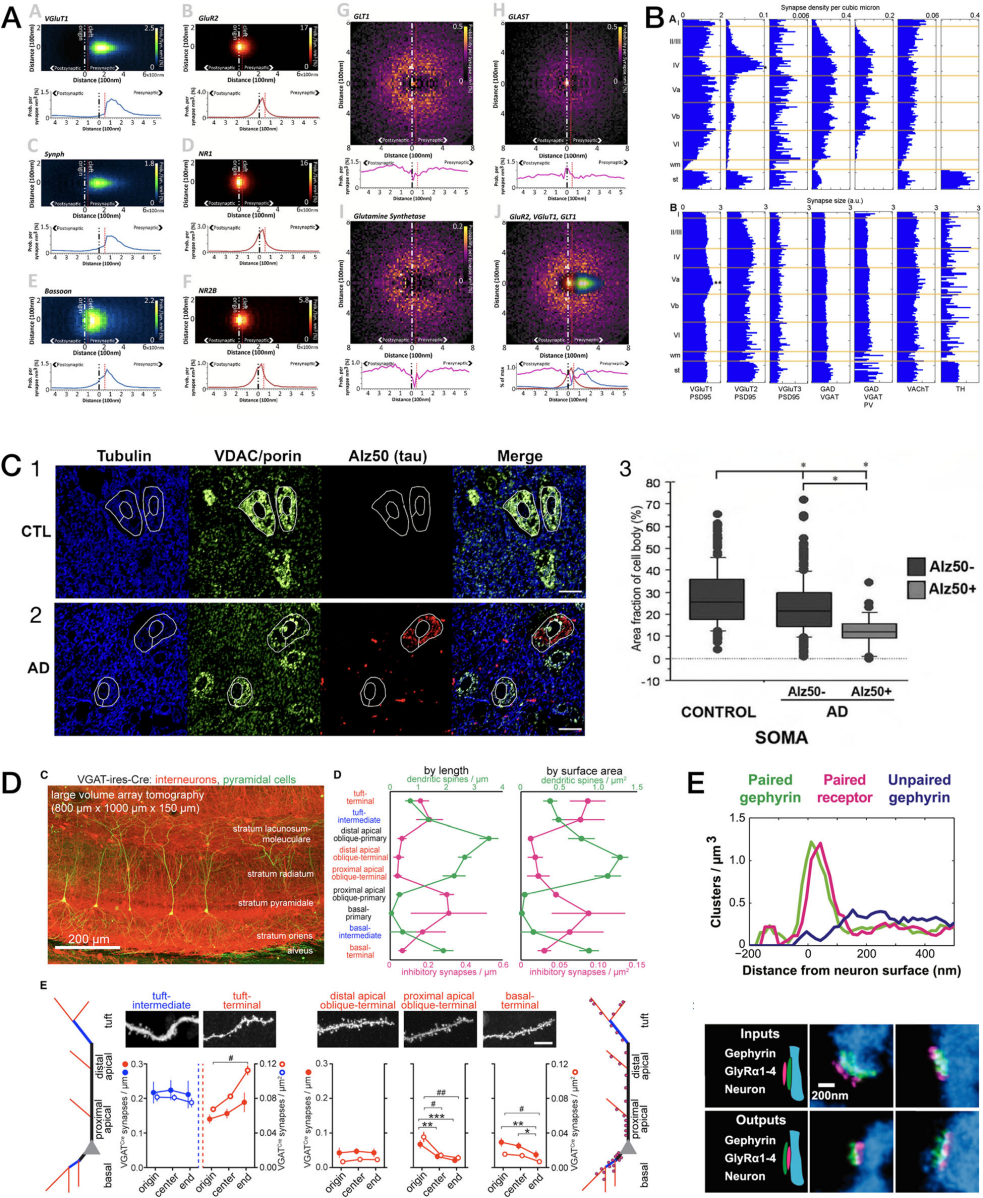
Quantitative analysis of AT images. **a** Nanoscale localization of multiple synaptic proteins along transynaptic axis, generated from 36,977 individual mouse cortical synapses (from Fig. 4 in [93]). **b** Variations with depth of volume density and size of seven molecular synapse types in mouse somatosensory cortex (from Fig. 5 in [85]). **c** Somatic mitochondrial distributions are disrupted in both pTau+ and pTau− neurons of superior temporal gyrus from Alzheimer disease (*AD*) vs control (*CTL*) human brains (from Fig. 5 in [83]). **d** Structured spatial patterning of inhibitory synapses onto mouse CA1 pyramidal cell dendrites (from Fig. 2 in [27], Copyright (2016), with permission from Elsevier). **e** Discrimination of input vs output glycinergic synapses to/from narrow-field amacrine cells by STORM fluorescence nanoscale AT (from Fig. 6 in [63], Copyright (2015), with permission from Elsevier)

The measurement of individual synapses in the central nervous system (CNS) neuropil has been one of the principal applications of AT to date. With fast widefield capture using an NA = 1.4 objective at the diffraction limit, FM-AT images are ideally sampled at about 100 nm pixel size. If sections are cut at a typical thickness of 100 nm, the resulting voxel volumes of 1 aL (attoliter) are well suited to resolving CNS synapses and their separate presynaptic and postsynaptic elements. As established by EM measurements, such synapses typically have total volumes ranging between 5 and 200 aL and are situated within an average neuropil volume of approximately 1000 aL. The advantages of FM-AT over whole-mount fluorescence microscopy for synaptomic applications are starkly evident when one considers that the AT’s tenfold improvement in Z-axis resolution combines with a twofold improvement in lateral resolution to improve volumetric resolution by a factor of at least 5 × 2 × 2 = 20. While super-resolution whole-mount fluorescence methods may enable certain synaptomic measurements comparable to those offered by FM-AT (e.g., [69]), they do so at the cost of much slower image acquisition and remain subject to depth-dependence artifacts, and thus may not be suitable for imaging at the larger volume scales required for many synaptomic and connectomic purposes. Excellent results from fast, widefield FM-AT sampling of mouse CNS synapses are exemplified in Figs. 3c–e, 4a–c, 6b, 7c, and 9a, b.

## What limits AT specimen size?

AT specimens prepared as a single ribbon array (e.g., Fig. 1) might comprise 50 serial sections, each perhaps 2 mm wide by 0.5 mm long by 100 nm thick, yielding a total specimen volume 2 × 0.5 × 0.005 mm in X, Y, and Z. The logistics of serial sectioning and fluorescence imaging naturally encourage acquisition of such “flattened” specimen cuboids, where the least dimension lies along the Z-axis. The single ribbon volume in this example would comprise 5 nL (nanoliters). A total specimen volume of 5 nL may sound small, but note that one nanoliter of mouse cortical gray matter comprises approximately one million synapses! Many of the synaptomic FM-AT volumes illustrated here and in other publications involve only a fraction of a nanoliter, yet represent very large numbers of reliable single-synapse observations. EM-AT volumes may be even smaller: the spectacular EM-AT images rendered in Fig. 5c resulted from mid-resolution imaging of approximately 25 nL and high-resolution imaging of only 0.08 nL of mouse cortex. The acquisition and processing of AT images at the 5 nL scale with today’s fastest technologies may take a few days (for FM-AT) to several weeks (for EM-AT) and entail the reasonable data storage costs associated with a few to a few dozen terabytes.

For the analysis of larger structures, such as complete neuronal dendrites and local circuits, it may be desired to image much larger volumes, on the order of 1000 nL (i.e., one microliter or 1 cubic millimeter). Larger and thicker high-resolution AT volumes necessarily require collecting and imaging larger numbers (i.e., thousands or tens of thousands) of serial sections collected using some form of automation (a tape collector or a multi-ribbon collection robot like that illustrated in Fig. 8a, b). The upper limits to practical AT specimen size are then essentially economic: the costs in hardware, reagents, image acquisition time, data storage requirements, and image processing and analysis loads. All the recurring costs of AT scale approximately linearly with specimen volume and at present must be considered expensive. Figure 3a, b represent the largest AT volumes so far reported in the literature, comprising 175 nL and 50 nL, respectively. These efforts have required extensive custom microscope engineering and software development and person-years of experimental effort. The acquisition and processing of AT images at the 1000 nL scale with these evolving technologies may take several weeks (for FM-AT) to many months (for EM-AT) and entail the daunting data storage costs presently associated with petabyte scale requirements. The upper limits to practical AT specimen size are thus essentially economic: the costs in hardware, image acquisition time, data storage requirements, and image processing and analysis loads. Thus, imaging at the 1000 nL scale looms as a formidable and expensive challenge today. Nonetheless, assuming continued advances in both imaging tool speed and data processing and storage economics, it is reasonable to imagine that 1000 nL and still larger volumes may become much more practical in coming years.

Large differentials in cost between FM-AT image acquisition (fast and inexpensive) and EM-AT imaging (much slower and more expensive) suggest consideration of hybrid FM/EM sampling strategies for many types of project. The FM/EM-AT modality allows for the imaging of relatively large volumes by FM-AT to be followed by the sparse sampling of smaller subvolumes by EM-AT, capturing many key advantages of both AT modes at reasonable cost. Information gained from the sparse FM/EM-AT volumes can be used to more deeply and rigorously interpret the larger, purely FM-AT volumes. The power of this approach has been demonstrated by recent analyses of mouse CNS synapse populations [27, 28, 31] and it seems likely that buttressing the strengths of FM-AT with sparse EM-AT in this way will help to manage the costs of data acquisition handling associated with many future large-scale AT projects.

## What makes AT especially suitable for sequential multiplexing?

Rapid increases in readily accessible computational power, digital storage capacities, and fluorescence molecular assay strategies have kindled substantial interest in sequential multiplexing methods for molecular microscopy. The 2007 introduction of AT [17] demonstrated sequential multiplexing to read out 11 molecular markers and quantitative stability across six sequential rounds of differential staining and imaging. A 2010 publication [37] demonstrated quantitative imaging of 18 markers by six sequential rounds of fluorescence imaging.

Sequential molecular multiplexing methods based on in situ sequencing and decoding of DNA-bar-coded in situ hybridization probes or bar-coded antibodies are expected to soon advance multiplexing far beyond present practices for both AT and non-AT molecular imaging [70, 71]. With growing excitement about such “bar coding” methods, however, attention must be paid to fundamental limits. Sequential multiplexing requires repeated rounds of imaging interspersed over time by labeling and rinsing washes, with some form of probe elution or bleaching, followed by imaging processing steps to bring images from the sequential rounds into spatial register. Any physical instability during sequential imaging rounds will complicate cross-round image registration and place limits on multiplexing possibilities. Three-dimensional specimens, even when reinforced by polymer gel fixation, are subject to deformation over time and especially when subjected to different staining, wash, or elution solutions. Such instabilities tend to worsen when tissue proteins and lipids are replaced by water, as in the various tissue clearing and expansion methods now coming into use. Because deformations of thick tissue samples are likely to include anisotropic and unpredictable components, imperfect registration of sequential images will limit multiplex image interpretation and compromise prospects for single-molecule multiplexing (e.g., for in situ sequencing or probe decoding), particularly at high target labeling densities. The extreme physical stability of AT specimens, where resin-embedded ultrathin sections are tightly bonded to a solid substrate, decisively eliminates such registration difficulties.

## What makes FM-AT especially quantitative?

Fluorescence AT offers unique opportunities to quantify fluorescence signals independent of depth within a tissue specimen. When imaging whole-mount tissue specimens, fluorescence readout tends to fall off with increasing focal depth due to increasing light scattering, absorption, and optical aberrations. When whole-mount specimens are labeled by immunostaining, quantitative analysis is further compromised by stain reagent diffusion limits. All such effects are eliminated by AT physical sectioning, where each section—regardless of depth with the original specimen—is stained and imaged identically in a planar format.

## What image processing does AT require?

The processing of AT images usually begins with flat-field correction [72] to correct for variations in image brightness across individual image tiles due to microscope illumination or detection characteristics. Images of ultrathin AT sections are then ideal for restoration of high spatial frequencies (i.e., fine image details) by optimal deconvolution methods such as Richard-Lucy that assume a planar specimen geometry [32, 67]. Following such two-dimensional image restoration steps, precise computational alignment of two-dimensional serial section images to reconstruct a three-dimensional volume image is fundamental to all forms of AT [73–75]. For larger volumes, it is also usually necessary to stitch together multiple camera image tiles to compose seamless two-dimensional image section mosaics. Small geometric distortions typically occur during sectioning and section collection and generally require non-rigid transformation of individual tiles and sections to achieve sub-pixel-accurate alignment. When multiple images acquired across multiple image acquisition sessions (as in Fig. 1b) or across multiple microscopes (as in Fig. 1d) rigid and/or non-rigid transformations may also be required to register all two-dimensional image planes into a common pixel space.

As with all forms of serial-section microscopy, it is occasionally necessary to deal gracefully with section defects such as wrinkles, folds, tears, surface contamination, or even the occasional missing section. Since such defects are ordinarily rare and sparse, the data loss per se is usually not overly troublesome, but their presence may perturb high-quality alignment unless recognized and corrected. Finally, when integrating fluorescence and electron AT modalities for FM/EM-AT, image processing must deal efficiently with the wide differential of pixel sizes appropriate to fluorescence (~ 100 × 100 nm) and electron (~ 3 × 3 nm) image acquisition. Moreover, even though AT makes registration of FM and EM images in the Z dimension trivial, useful registration of FM and EM images in X and Y axes must be accurate to a scale set by the very small EM pixel size and may require highly specialized image alignment methods [31, 76]. All of these requirements add up to substantial demands for computational resources, such that cluster or cloud computing and web-based solutions become most appropriate [77–79]. Computational automation of complex AT image processing workflows becomes a virtual necessity as AT imaging is scaled to larger tissue volumes.

## How are AT images analyzed?

Specific protocols for analysis of AT images naturally depend on the nature of the specimen and the biological question addressed. The prevalent AT application to date has been in synaptomics, where AT is prized for reliable resolution, detection, and measurement of synapses crowded into brain tissue context [17, 27, 28, 30, 31, 35, 55, 63, 80–98]. Figures 3, 4, 5, 6 and 8 illustrate a small selection of results of synaptomic and other published analysis protocols; the publications cited in the corresponding figure legends should be consulted for details of each particular illustration and analysis. The free, general-purpose NIH ImageJ software platform, its Fiji distribution package [99] and its TrakEM2 companion [100] provide excellent starting points for AT image analysis, as this platform is flexible and very suitable for the processing and analysis of high-dimensional AT images. Numerous commercial software solutions may better suit specific analysis scenarios, however, and analysis of the larger, high-resolution, high-content AT image datasets will necessarily tend toward requiring web- and cloud-based solutions to data storage, processing, and analysis challenges. Machine learning tools, including deep convolutional networks, are now revolutionizing all forms of volumetric image analysis, AT analysis included [76].

As a volumetric imaging modality, AT poses both challenges and opportunities for the computational segmentation of biologically meaningful three-dimensional objects, and for quantification of such objects. In the realm of circuit neuroscience, such image analyses have focused on detection of synapses, profiling of diverse synapse populations, and tracing of axons and dendrites. The high-dimensional molecular discrimination capacities realized in AT also pose exciting new opportunities to fathom the brain’s intricate molecular architectures, but with these will certainly come new challenges to data analysis.

## What lies ahead for AT?

Improved materials and methods for AT are rapidly increasing the power of AT and shrinking the technical difficulty that has so far limited applications. Ongoing materials engineering aims to improve the scope and sensitivity of AT molecular analysis: (1) resin chemistry to improve label access to embedded tissue proteins; (2) new protein labeling reagents, such as array-screened monoclonals, camelid antibodies, nanobodies, and recombinant immunoglobulin fragments to improve protein detection; (3) new organic and biologic fluors, fluorescent nanoparticles, cathodoluminescent tags, and DNA-barcoded antibody tags; and (4) new resin and probe chemistries to enable in situ mRNA hybridization with immunolabeling at AT resolution. As these new methods and others advance, technical and economic barriers to AT application should fall and enable the development of many new AT applications inside and outside of neuroscience. As AT methodologies become more routine and less expensive, application areas may eventually even grow to include clinical pathology.

The economic obstacles to large-scale AT should fall. With successes of ongoing AT process and tool engineering, commercialization advances, and continuation of the “Moore’s Law” deflation of computing costs, it can be anticipated that AT imaging of the microliter-scale volumes needed for local circuit connectomics and certain other tissue analysis challenges may eventually become routine. For large-scale, high-resolution digital microscopy, image acquisition times quickly become the rate-limiting step. (Several microliter-scale volumetric EM projects now under way envision image acquisition times on the order of months to years!) Here, AT offers substantial speed advantages in comparison to other comparable methods, because of AT’s high optical imaging efficiency. The engineering prototype illustrated in Fig. 8c acquires 1 aL, super-diffraction FM-AT voxels at an overall net rate well in excess of 10 million per second. The multibeam scanning electron microscope (mSEM) offers the prospect of acquiring EM-AT volume images at rates approaching one billion 0.001 aL EM-AT voxels per second.

Perhaps the most vibrant near-term growth in AT applications will build on the rapid advance of mRNA sequencing technologies. A flood of deep, single-cell transcriptomic data has ushered in the prospect of classifying neurons and other tissue cells into taxonomies comprising modest numbers of relatively discrete cell types, each complete with its own distinctive “parts list” of protein products predicted from gene expression patterns. At the simplest level, single-cell transcriptomic data and cell-type taxonomies will guide the selection of AT antibodies to more deeply and efficiently explore cell-type-specific molecular mechanisms in tissue architectural context. The AT superlatives of resolution, volume scalability, and molecular multiplexing are also likely to prove excellent fits to the challenges of cross-validating transcriptomic taxonomies to other dimensions of cell type differentiation (e.g., anatomy, physiology, proteomics, connectomics, or synaptomics). The prospects for AT cross-validation of cell-type and synapse-type taxonomies will likely grow even faster if resin chemistry developments enable some form of highly multiplexed RNA-FISH imaging compatible with existing immunofluorescence imaging capacities. In any case, detection of proteins predicted by mRNA transcript detection should strongly advance cross-validation of molecular taxonomies and offer new and fundamental insights into quantitative transcript–protein relationships. The strengths of AT imaging appear to match the needs of an approaching post-transcriptomic tissue science era very nicely indeed!
